# Characterization of the mammalian family of DCN-type NEDD8 E3 ligases

**DOI:** 10.1242/jcs.181784

**Published:** 2016-04-01

**Authors:** Matthew J. Keuss, Yann Thomas, Robin Mcarthur, Nicola T. Wood, Axel Knebel, Thimo Kurz

**Affiliations:** Medical Research Council Protein Phosphorylation and Ubiquitylation Unit, College of Life Sciences, University of Dundee, Dundee DD1 5EH, UK

**Keywords:** Ubiquitin, NEDD8, Cullin-RING ligases, CRL, SCCRO, DCUN1D

## Abstract

Cullin-RING ligases (CRL) are ubiquitin E3 enzymes that bind substrates through variable substrate receptor proteins and are activated by attachment of the ubiquitin-like protein NEDD8 to the cullin subunit. DCNs are NEDD8 E3 ligases that promote neddylation. Mammalian cells express five DCN-like (DCNL) proteins but little is known about their specific functions or interaction partners. We found that DCNLs form stable stoichiometric complexes with CAND1 and cullins that can only be neddylated in the presence of a substrate adaptor. These CAND–cullin–DCNL complexes might represent ‘reserve’ CRLs that can be rapidly activated when needed. We further found that all DCNLs interact with most cullin subtypes, but that they are probably responsible for the neddylation of different subpopulations of any given cullin. This is consistent with the fact that the subcellular localization of DCNLs in tissue culture cells differs and that they show unique tissue-specific expression patterns in mice. Thus, the specificity between DCNL-type NEDD8 E3 enzymes and their cullin substrates is only apparent in well-defined physiological contexts and related to their subcellular distribution and restricted expression.

## Abstract (precis)

**Highlighted Article:** The mammalian DCN-type NEDD8 E3 ligases (DCNL1–5) have overlapping and unique functions that might be related to their subcellular distribution and are mostly governed by their unique N-termini.

## INTRODUCTION

Ubiquitin is a small (8 kDa) signaling protein that regulates most cellular activities. The majority of ubiquitin's functions require its linkage to other proteins through isopeptide bonds. This is mediated by the sequential action of three enzyme families, termed E1, E2 and E3 (Hershko and Ciechanover, 1998). E3 enzymes are much more numerous (∼600 proteins) than E1 (2 proteins) and E2 enzymes (∼40 proteins), reflecting their role as substrate specificity factors of the reaction (Hershko and Ciechanover, 1998). By far the largest class of E3 enzymes is formed by the cullin-RING ligase (CRL) family. These are modular E3s built around a heterodimeric catalytic scaffolding complex that consists of a small RING-finger protein (either RBX1 or RBX2) bound to the C-terminus of a cullin protein. The N-terminus of cullin proteins can interact with many different substrate-specificity modules that recruit substrates, whereas the RING finger protein interacts with ubiquitin-charged E2 enzymes (Petroski and Deshaies, 2005). Mammalian cells contain eight cullin proteins, Cul1, Cul2, Cul3, Cul4A, Cul4B, Cul5, Cul7 and Cul9/Parc8 (Sarikas et al., 2011) and although only a limited number of CRL complexes have assigned substrates, it is estimated that ∼300 CRL complexes exist in humans (Enchev et al., 2015).

The assembly and activity of CRLs is regulated through reversible conjugation of NEDD8, a ubiquitin-like protein, to a conserved lysine residue in the cullin backbone (Lammer et al., 1998; Liakopoulos et al., 1998; Pintard et al., 2003; Lyapina et al., 2001; Bornstein et al., 2006; Cope et al., 2002). NEDD8 modification activates CRLs by inducing structural flexibility of the cullin C-terminus, allowing RBX1 and RBX2 to adopt productive conformations for the transfer of ubiquitin onto substrates (Duda et al., 2008). Neddylation and de-neddylation of cullins are also important to regulate CRL composition, as only de-neddylated complexes can interact with the exchange factor CAND1 that regulates the release of old and the association of new substrate adaptors to cullin–RING core complexes (Goldenberg et al., 2004; Liu et al., 2002; Zemla et al., 2013; Zheng et al., 2002; Pierce et al., 2013; Wu et al., 2013). Like ubiquitin, NEDD8 becomes conjugated to its cullin substrates by E1, E2 and E3 enzymes (Kurz et al., 2005; Rabut and Peter, 2008; Rabut et al., 2011). The single E1 NEDD8-activating enzyme (NAE) is a heterodimer of two proteins, APP-BP1 (also known as NAE1 and ULA1) and UBA3 (Walden et al., 2003). Two NEDD8-conjugating E2 enzymes are encoded by UBE2M and UBE2F (Huang et al., 2009). In addition to being ubiquitin E3s, RBX1 and RBX2 are also NEDD8 E3s that transfer NEDD8 onto the cullins they are bound to, but they require auxiliary E3 factors to direct NEDD8 towards the right lysine residue (Scott et al., 2010). These factors are encoded by proteins of the DCN1 (defective in cullin neddylation 1) family. In lower organisms, single DCN1 homologs exist that promote the neddylation of all cullins (Kurz et al., 2005, 2008), whereas multiple DCN1 molecules are encoded by the genomes of higher organisms. Human cells, for example, express five DCN1-like proteins termed DCNL1–DCNL5 (also named DCUN1D1–5 for defective in cullin neddylation 1 domain-containing protein 1–5 or SCCRO1-5) (Bommelje et al., 2014; Sarkaria et al., 2006; Meyer-Schaller et al., 2009; Kim et al., 2008; Huang et al., 2014). These DCNLs have distinct N-terminal domains, but share a conserved C-terminal potentiating neddylation (PONY) domain. The PONY domain directly binds to cullins through invariant residues, called the DAD patch (D226, A253, D259 in *Saccharomyces cerevisiae* Dcn1), whereas the function of the variable N-terminal domains are largely unclear (Kurz et al., 2005). The N-terminus of yeast Dcn1 encodes a ubiquitin-binding UBA domain, which is also present in the human DCNL1 and DCNL2 isoforms. Although conserved, the UBA domain is not required for DCN1s neddylation activity *in vitro* or *in vivo* and its function remains to be determined (Kurz et al., 2008; Wu et al., 2011). The regulation of DCNL activity in general appears to be important, as DCNL1 is highly amplified in various tumors where it acts as an oncogene (squamous-cell-carcinoma-related oncogene) (Broderick et al., 2010; Sarkaria et al., 2006) and DCNL5 (DCUN1D5) was also recently reported to be overexpressed in some oral and lung squamous cell carcinomas (Bommelje et al., 2014). Consistent with these observations, inhibition of neddylation by a small molecule drug (MLN4924) shows promise in clinical trials for the treatment of hematological malignancies (Soucy et al., 2009).

Whereas DCNL1 and DCNL2 contain N-terminal UBA domains, DCNL3, DCNL4 and DCNL5 contain unique N-termini that are predicted to be unstructured. In general, the N-termini are thought to govern the subcellular localization of DCNs or their cullin specificity. However, comprehensive evidence in support of this hypothesis is missing, except for DCNL1 and DCNL3 (Wu et al., 2011; Meyer-Schaller et al., 2009).

Here we have explored the specificity and interactions of the different mammalian DCNL homologs. We find that in cells all DCNLs interact strongly with most cullins and CAND1, a known regulator of CRLs that is required to exchange substrate adaptors (Liu et al., 2002; Zheng et al., 2002). We further find that the five mammalian DCNLs are widely expressed in tissue culture cells, but differ in their subcellular localization patterns. The expression of DCNLs in animal tissue is more restricted, where DCNL1 seems to be the only broadly expressed DCNL. In tissue culture cells, none of the DCNLs shows specificity towards a cullin subtype, supporting a model whereby all or most DCNLs contribute to the overall neddylation levels of cullins. However, it also suggests that DCNLs are not simply redundant, but instead neddylate distinct subpopulations of cullins. One striking example is the non-redundant contribution of DCNL1 and DCNL5 to the neddylation of cullin4A. Our findings thus suggest that although DCNLs appear to indiscriminately neddylate cullins, they maintain unique functions that are not redundant with other DCNLs.

## RESULTS

### DCNLs interact with cullins and CAND1

**Fig. 1.**
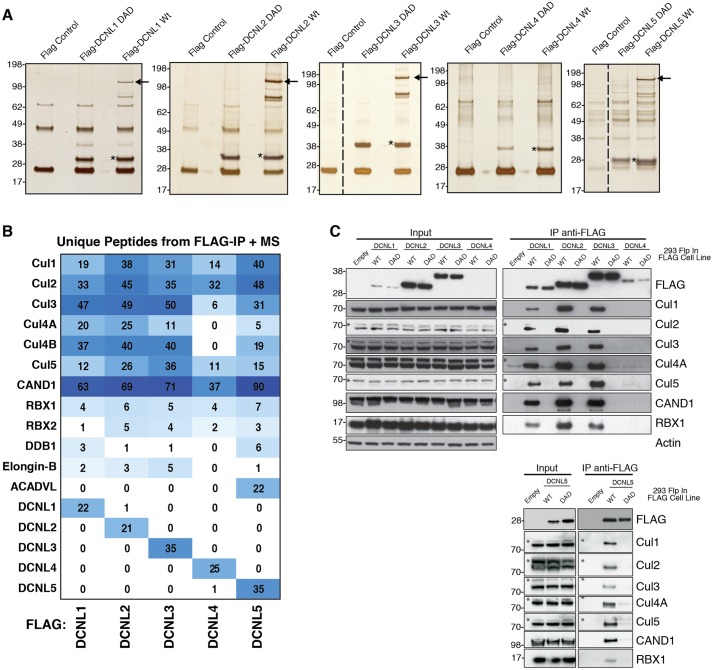
**Characterization of DCNL interactors.** (A) Silver-stained gels of FLAG immunoprecipitations from HEK293 cells stably expressing wild-type (WT) or cullin-binding-deficient DAD patch mutants of all mammalian DCNLs (DCNL1–5). Asterisks denote FLAG–DCNL proteins. WT, but not DAD mutants, specifically co-immunoprecipitate proteins that are not present in the empty FLAG or control, including a large protein with molecular mass >98 kDa (arrow). (B) Heatmap of DCNL interactors identified by mass spectrometry of immunoprecipitations in A. Interactors with >3 unique peptides detected are plotted. All DCNLs co-immunoprecipitate most cullins, as well as cullin-associated proteins RBX1, RBX2 (RNF7), the Cul4A adaptor protein DDB1 and the Cul2/5 adaptor elongin B (TCEB1). The strongest interactor of all DCNLs is CAND1, the CRL substrate adaptor exchange factor. (C) Immunoprecipitation of FLAG–DCNL1, 2, 3 and 4 (top panels) and FLAG-DCNL5 (bottom panels) from stably expressing HEK293 cells followed by western blot analysis. The slower migrating of the two cullin-reactive bands is the neddylated form (asterisk). Only WT DCNLs co-immunoprecipitate mostly non-neddylated cullins, CAND1 and RBX1. Expression levels of FLAG–DCNL4 were too low in this experiment to detect co-immunoprecipitating proteins.

Using HEK-293 cell lines stably expressing N-terminally FLAG-tagged isoforms of each DCNL, we determined their interaction partners by mass spectrometry after FLAG-immunoprecipitation (Kurz et al., 2008) (Fig. 1). Using SDS-PAGE gels followed by silver staining (Fig. 1A,C), we detected unique interaction patterns for each DCNL, with the exception of one protein at >100 kDa, that was prominently present in all immunoprecipitates, but too large to be a cullin. All the specific interactors were lost when the DAD patch was mutated, suggesting that the interactions are mediated by cullins.

The mass-spectrometry analyses revealed that most DCNLs interacted with most cullin subtypes (Cul1, 2, 3, 4A, 4B and 5) with the exception of Cul7 and Cul9 (Fig. 1B). There was no readily apparent difference in the binding to the different cullins, except for DCNL4, which seemed to be overall binding less to Cul3 and did not bind to Cul4A or Cul4B (Fig. 1B). However, this apparent lack of interaction was probably a result of the very low expression level of FLAG–DCNL4 in our cell line compared with the other cell lines. This resulted in considerably less FLAG–DCNL4 being immunoprecipitated (Fig. 1C), which might have dropped the amount of co-precipitated Cul4A and Cul4B below the detection limit. Nevertheless, from these results we concluded that no DCNL has a readily apparent cullin preference in cells. This is in accordance with previously published *in vitro* data that showed that purified recombinant DCNLs can bind to all cullins with only slightly different affinities (Monda et al., 2013). Our results now suggest that in cells, DCNLs are indeed capable of binding to most, if not all, cullins indiscriminately. Interestingly, when we went on to confirm the identified interactions by western blotting (Fig. 1C) we found that DCNLs only interacted with non-neddylated cullins, strongly suggesting that they are released from the cullin complexes once they are neddylated. Consistent with the low expression levels of our FLAG–DCNL4 cell line, we were unable to confirm by western blot the interactions between FLAG–DCNL4 and the binding partners identified by mass spectrometry.

In the mass spectrometry analysis we found very few additional proteins outside of cullins that specifically bound to DCNLs. DCNL1, 2, 3 and 5 also interacted with other regulators or subunits of cullin-RING ligases such as RBX1, RBX2, elongin B (Cul2 substrate adaptor) and DDB1 (Cul4A substrate adaptor). The only non-cullin related interactor we identified was the mitochondrial protein ACADVL that bound to DCNL5. We could not, however, independently confirm this interaction by western blotting, casting doubt on the validity of this interaction (Fig. S1).

**Fig. 2.**
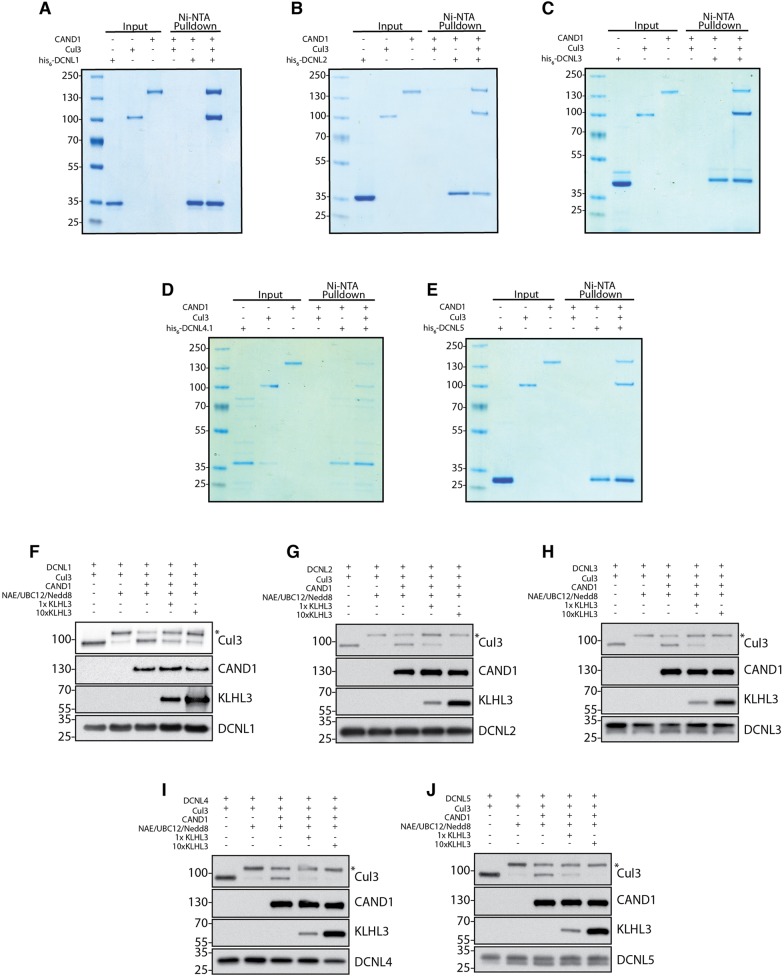
**DCNLs forms a stable complex with cullin and CAND1.** (A–E) His_6_–DCNL1, 2, 3, 4 and 5 were bound to magnetic Ni-NTA beads, incubated for 20 min at 4°C with CAND1 or Cul3–CAND1 complexes and washed with 10 mM imidazole to reduce nonspecific binding. Bound proteins were eluted with 300 mM imidazole and separated by SDS-PAGE followed by Coomassie staining. CAND1, Cul3 and DCNLs form a stoichiometric complex (lane 7) and CAND1 does not bind to any His_6_–DCNL in the absence of Cul3 (lane 6). (F–J) *In vitro* neddylation reactions of Cul3 show inhibition by CAND1 and rescue with substrate adaptor KLHL3. Each reaction contained 1 µM Cul3 and 1 µM DCNL and as indicated 1 µM CAND1, 1 µM KLHL3 (1×), or 10 µM KLHL3 (10×). Neddylation was induced by addition to a final concentration of 34 µM NEDD8, 4 µM UBE2M, and 0.2 μM NAE in 50 mM Tris-HCl pH 8 with 0.15 mM ATP, 1.5 mM MgCl_2_ and 20% glycerol. Reactions were performed for 2 min at 30°C and quenched by the addition of SDS loading buffer. Samples were resolved by SDS-PAGE and processed for immunoblotting with the indicated antibodies. Cul3 is readily neddylated in the absence of CAND1 as seen from the ∼10 kDa band shift in lane 2 (asterisk). Addition of CAND1 inhibits the neddylation reactions, and inhibition is relieved upon the addition of Cul3 substrate adaptor KLHL3.

Most strikingly, we detected a very strong interaction of all DCNLs with CAND1 (Fig. 1B). This was surprising, as CAND1 only binds to non-neddylated cullins and prevents their neddylation when bound (Liu et al., 2002; Zheng et al., 2002). Based on peptide counts, CAND1 was the strongest interactor for all DCNs, and by size, it fit the large protein that we had readily identified on silver-stained gels (Fig. 1A). A mutation of the DAD patch on DCNLs abolished binding to all cullins, but also to CAND1, suggesting that the interaction is bridged by cullins (Fig. 1C). Indeed, all DCNLs were able to form stable stoichiometric heterotrimeric complexes *in vitro* with recombinantly expressed CAND1 and the cullin Cul3−Rbx1 (Fig. 2A–E). Formation of the complexes *in vitro* was dependent on the presence of Cul3−Rbx1, and we could not detect a direct interaction between any DCNL and CAND1 (Fig. 2A–E). Thus, DCNLs can form stable stoichiometric complexes with cullins and CAND1 that are bridged by the cullin protein.

Given that CAND1–cullin complexes are resistant to neddylation, we next tested if this was also true for CAND1–Cul3–DCNL complexes. We could show *in vitro* that the presence of CAND1 strongly inhibited the neddylation of Cul3–Rbx1 irrespective of the presence of any DCNL in the complex (Fig. 2F–J). These results posed the question why such complexes of CAND–cullin–DCNL would form in the first place. One possibility was that they represent reservoir cullin complexes that are inactive, but primed for neddylation and thus rapid activation when needed. As active complexes need to bind to substrates, we reasoned that the inactive complexes might become activated in the presence of a substrate adaptor. Indeed, the addition of stoichiometric amounts of the Cul3 substrate adaptor KLHL3 to the *in vitro* reaction overcame the CAND1-dependent inhibition of neddylation (Fig. 2F–J). It is thus highly plausible that heterotrimeric CAND1–cullin–DCNL complexes exist in the cell to allow rapid activation by neddylation as soon as they encounter substrate adaptor complexes.

### DCNLs differ in their subcellular localization and expression profiles

**Fig. 3.**
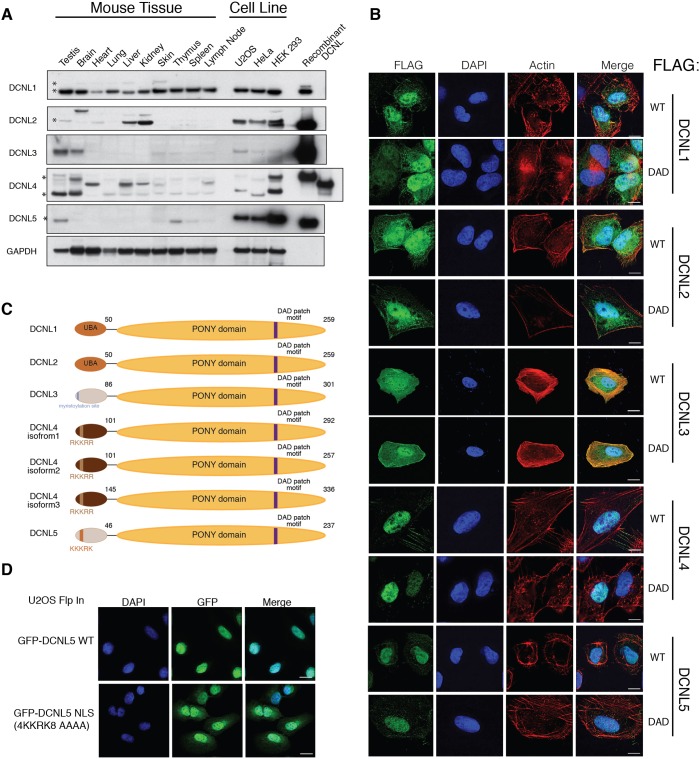
**Tissue-specific expression and subcellular localization of DCNLs.** (A) DCNLs have unique tissue expression profiles and upregulation in cancer cell lines. Western blot analysis of mouse tissue lysates and cancer cell lysates (30 μg each) and where indicated recombinant protein (50 ng). DCNL1 is widely expressed in all tissue samples as well as cancer cell lines. DCNL2 expression is more restricted with highest expression in the brain, liver and kidneys. At least one of the three isoforms of DCNL4 is expressed in most tissue, albeit at varying levels. DCNL3 expression is restricted to testis and brain with weak expression in cancer cell lines. DCNL5 has low expression in testis, skin, thymus and spleen but is drastically upregulated in the three cancer lines. (B) Subcellular localization of DCNLs is independent of cullin binding. U2OS cells expressing N-terminally FLAG tagged DCNL1–5 were analysed by indirect immunofluorescence with an anti-FLAG antibody, rhodamine-conjugated Phalloidin (actin) and DAPI staining (scale bars: 10 µm). DCNL1 and DCNL2 are localized throughout the cell in both the cytoplasm and nucleus. DCNL3 is located throughout the cell, but also at the plasma membrane. DCNL4 and DCNL5 are restricted to the nucleus. Mutation of any of the DCNLs DAD patch domains does not cause a change in subcellular localization, suggesting DCNLs localization is independent of cullin binding and instead determined by their unique N-terminal domain. (C) Diagram of each DCNL family member depicting their conserved C-terminal PONY domain and specific N-terminal domain. Depicted on DCNL3 is a myristoylation site and depicted on DCNL5 is a nuclear localization sequence (NLS). (D) Immunofluorescence analysis of U2OS cells stably expressing either GFP–DCNL5 WT or GFP–DCNL5 with mutations in the NLS (scale bar: 10 µm). Mutation of the NLS sequence results in a delocalization of some DCNL5 into the cytoplasm.

Based on our mass spectrometry results, all DCNLs are able to interact with most cullins. We thus next tried to understand if they are fully redundant or also have unique functions. We first determined their expression profiles in mouse tissue and tissue culture cells. Using specific antibodies for each family member (Fig. S2), we found that all DCNLs are well-expressed in the three tissue culture cell lines we tested (HEK-293, U2OS and HeLa) (Fig. 3A). DCNL1 and DCNL2 are expressed to similar levels in all three cell lines. DCNL3 expression is comparatively low, but it is best expressed in U2OS cells and less abundantly in HeLa and HEK-293 cells (Fig. 3A). DCNL4 is predicted to have at least three splice variants (29 kDa, 34 kDa, 38 kDa), and we can detect at least two of these isoforms in tissue culture cells to relatively high levels, but most strongly in HEK-293 cells (Fig. 3A). Strikingly, DCNL5 is the most strongly expressed DCNL in all cell lines tested, suggesting that it might have a major role in proliferating cells (Fig. 3A).

This general expression is in stark contrast to DCNL expression in mouse tissue. Here, DCNL5 was the least expressed of all DCNLs (Fig. 3A). DCNL1, by contrast, was the most widely expressed isoform and could easily be detected in all tissues (Fig. 3A). DCNL2, although very similar in sequence to DCNL1, was not expressed as widely and mostly found in liver and kidney and, as what seemed to be a highly post-translationally modified form, in brain (Fig. 3A). Expression of DCNL3 was mostly restricted to testis and brain. The expression of different DCNL4 isoforms was more widely detectable, but most strongly in testis, brain, heart, liver and kidney (Fig. 3A). DCNL4 was predominantly expressed as the 34 kDa isoform, except in testis, where the smaller 29 kDa isoform was predominant, and in brain, where the 38 kDa and the 29 kDa isoforms were expressed to approximately equal levels (Fig. 3A). DCNL5 was only detectable at low levels in testis, skin and immune tissues (thymus, spleen and lymph nodes), with the highest expression in thymus and testis, suggesting that DCNL5 has unique functions in these cells (Fig. 3A; Fig. S3).

DCNL1 is the evolutionarily oldest DCN isoform and most closely related to the single homologs found in lower organisms, and it might therefore also function as the main DCNL for cullin complexes in mammalian cells (Kurz et al., 2005). However, DCNL1 cannot be solely responsible for the neddylation of all cullins, as DCNL1 knockout mice are viable, which would not be expected if all cullins were affected by the deletion of DCNL1 (Huang et al., 2011). Thus, the other DCNLs probably regulate the activity of certain subpopulations of CRL complexes. The restricted expression pattern of most DCNLs suggests that these functions could be tissue-specific. However, as no knockout animals for any of the other DCNLs exist, we decided to investigate the potential specificities in tissue culture cells, bearing in mind that some of the specific effects might be masked because of the strong expression of most DCNLs in this experimental system.

Previous work using recombinant proteins and *in vitro* assays demonstrated that all recombinant DCNLs bind all cullins with only slightly different affinities and can promote their neddylation *in vitro* (Monda et al., 2013). Our immunoprecipitation analysis is less sensitive, and it is difficult to comment on the relative affinities in cells using our methodology. However, we do find that most DCNLs bind to all cullins in our analysis with the exception of DCNL4, which does not interact with Cul4A or Cul4B. Furthermore, all human DCNLs can rescue the neddylation defect of yeast DCN1 knockout cells when overexpressed (Meyer-Schaller et al., 2009), demonstrating that they are all capable of neddylating cullins, which we could confirm with our *in vitro* reconstitution of neddylation. We thus concluded that it is likely that distinct subcellular localization or binding partners, rather than different affinities for different cullin subtypes, would mediate any specificity in the system.

To investigate potential differences in subcellular localization, we utilized stably expressing FLAG–DCNL U2OS cell lines to determine their localization by indirect immunofluorescence with an anti-FLAG antibody. This revealed that although there is some overlap in their localization, DCNLs do have unique localization patterns (Fig. 3B). We found that DCNL1 and DCNL2 localize to both the nucleus and the cytoplasm of cells as previously described (Huang et al., 2011) (Fig. 3B). DCNL3 was present at the plasma membrane, in the cytoplasm and nucleus (Ma et al., 2008; Meyer-Schaller et al., 2009) (Fig. 3B), whereas both DCNL4 and DCNL5 were almost exclusively nuclear (Fig. 3B). As both DCNL1 and DCNL3 subcellular localization is governed by their respective N-termini, we reasoned that this might also be the case for DCNL4 and DCNL5 (Wu et al., 2011). Indeed, both their N-termini harbor a putative nuclear localization signal (NLS) (Bommelje et al., 2014) and its mutation in DCNL5 led to a diffusion of the protein into the cytoplasm (Fig. 3C,D). This mutant form of DCNL5 is, however, not entirely excluded from the nucleus, as it is small enough to passively diffuse through the nuclear pore (27.5 kDa) (Fig. 3D). Interestingly, mutation of the DAD patch motif, which disrupts cullin binding, does not change the localization of any DCNL, which demonstrates that the subcellular localization of DCNLs is independent of the interaction with cullins, and must be mediated by other determinants, possibly their unique N-termini (Fig. 3C).

### DCNLs do not display a strong cullin preference in cells

**Fig. 4.**
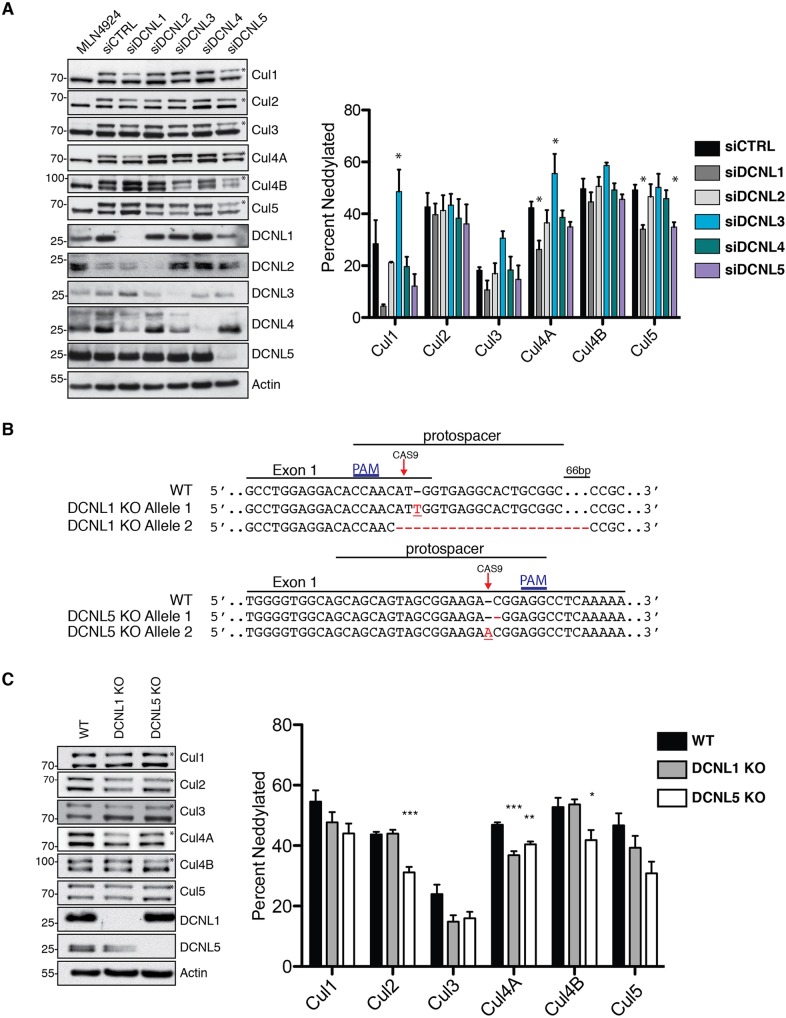
**Effects of DCNL downregulation on cullin neddylation.** (A) Knockdown of DCNLs by siRNA has only mild effects on the overall level of individual cullin neddylation. U2OS cells were treated with specific DCNL siRNAs or control siRNA for 72 h, and where indicated the neddylation inhibitor MLN4924 was added for 3 h at 3 μM. Following treatment, cells where harvested and processed for western blotting for the cullin family members to assess the fraction of cullin that was modified by NEDD8. Knockdown of DCNL1 reduces the amount of neddylated Cul1, 4A and 5, whereas knockdown of DCNL2 or DCNL4 had no effect on neddylation levels. Knockdown of DCNL3 increased Cul1 and Cul4A neddylation and DCNL5 knockdown reduced Cul4A and Cul5 neddylation levels. Adjacent graphs show the means±s.e.m. of quantified percentage of neddylated cullins. Statistical significance was determined by one-way ANOVA with Newman–Keuls multiple comparison test. *P≤0.05; *n*≥3. (B) Schematic of DCNL1 and DCNL5 sequences targeted by CRISPR/Cas9-mediated gene knockout with Protospacer adjacent motif (PAM) indicated (purple) and Cas9 cleavage site marked with an arrow. Depicted below each WT sequence are the mutations (in red) as determined by DNA sequencing. (C) Reduced cullin neddylation in U2OS knockout cells (individual clones) for DCNL1 and DCNL5 generated by CRISPR/Cas9. Whole cell lysates were prepared from WT, DCNL1 KO and DCNL5 KO and processed for SDS-PAGE and immunoblotting for cullin family members to assess the changes in the fraction of neddylated cullins upon loss of DCNL1 or DCNL5. DCNL1 knockout has the strongest effect on Cul4A neddylation and caused mild reduction of Cul3 and Cul5 neddylation. DCNL5 knockout caused reduced neddylation of all cullins tested with the greatest effect on Cul4A, Cul4B and Cul2. Adjacent graphs show the means±s.e.m. of quantified percentage of neddylated cullins. Statistical significance was determined by one-way ANOVA with Newman–Keuls multiple comparison test. *P≤0.05, **P≤0.01, ***P≤0.001; *n*≥3. Asterisks denote the slower migrating neddylated form of the two cullin-reactive bands.

The interaction data suggests that in tissue culture cells all DCNLs probably contribute to the neddylation of all cullins. To examine if this was the case and to determine the relative contribution of each DCNL to the overall steady-state neddylation level of Cul1, 2, 3, 4A, 4B and 5, we downregulated the expression of each DCNL by siRNA and determined the changes in cullin neddylation by western blot (Fig. 4A). As expected from our interaction data, downregulation of single DCNLs did not drastically affect the overall cullin neddylation levels, indicating that there is strong functional overlap between the different DCNL homologs in tissue culture cells (Fig. 4A). However, downregulation of some DCNLs affected some cullins more strongly than others. For example, although downregulation of DCNL1 seemed to slightly affect the neddylation of all cullins, it had a most prominent effect on Cul1 and Cul4A (Fig. 4A). This is similar to the downregulation of DCNL5, which also affected Cul1 and Cul4A (Fig. 4A). DCNL4 downregulation, by contrast, only slightly affected the neddylation of Cul5 and Cul4B (Fig. 4A). Curiously, downregulation of DCNL3 increased neddylation of Cul1, Cul3, Cul4A and Cul4B (Fig. 4A), which suggests that DCNL3 might in some instances inhibit cullin neddylation instead of promoting it (Huang et al., 2014). Thus, at least in tissue culture cells, there is not a single DCNL that is predominantly required for the neddylation of any one cullin. Instead, the different DCNLs appear to contribute to the overall neddylation pattern of most cullins.

However, given that siRNA downregulation might not remove all protein from the cell, it was possible that any remaining protein was sufficient to neddylate cullins and to thus mask more specific requirements. In order to explore this possibility, we generated knockout cell lines for DCNL1 and DCNL5 using the CRISPR/Cas9 method in U2OS cells (Ran et al., 2013; Munoz et al., 2014; Mali et al., 2013; Heigwer et al., 2014) (Fig. 4B). Similar to siRNA-mediated downregulation, a complete knockout of DCNL1 or DCNL5 did not entirely abolish the neddylation of any one cullin (Fig. 4C). However, it did more significantly affect the neddylation of most cullins compared with siRNA-mediated knockdown of DCNL1 and DCNL5 (Fig. 4A,C). Knockout of DCNL1 reduced the neddylation of Cul1, Cul3, Cul4A and Cul5, but left Cul2 and Cul4B unaffected (Fig. 4C). DCNL5 knockout, by contrast, affected all cullins tested (Fig. 4E). However, neither knockout affected the neddylation of any cullin to more than ∼25% of the parental cell line (Fig. 4C). The effects we see on the neddylation of different cullins does not directly correlate with the reported affinities in Monda et al. (2013) or our slight differences in interaction between cullins and DCNLs as analysed by mass spectrometry. However, given that the different affinities are ultimately very similar, it is not surprising that other factors govern which cullin is predominantly affected by a specific DCNL. Overall these results again highlight the fact that different DCNLs contribute to the steady-state neddylation level of many cullins, but they also suggest that distinct DCNLs are responsible for the neddylation of a given subpopulation of a cullin, because if they were entirely redundant, we would not expect to detect any effects on cullin neddylation upon loss of a single DCNL. Thus, DCNLs might have non-redundant functions with respect to certain cullin subpopulations and these could be mediated by differences in their subcellular localization.

### DCNL1 and DCNL5 independently contribute to cullin 4A neddylation, but only DCNL5 affects the DNA damage response

**Fig. 5.**
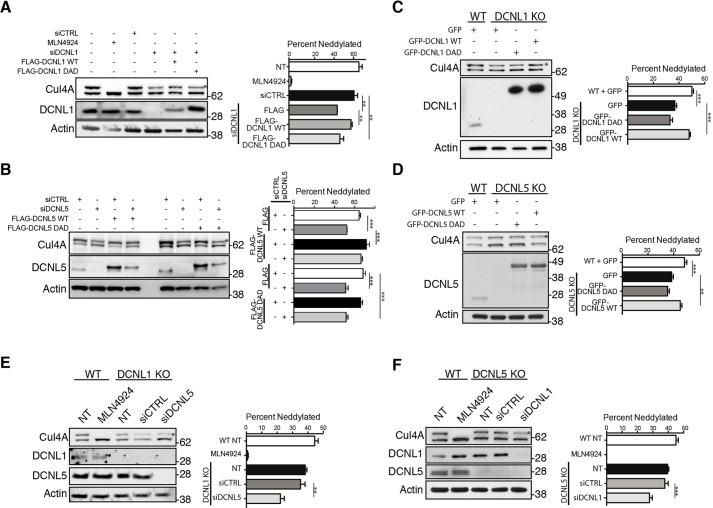
**DCNL1 and DCNL5 independently contribute to Cul4A neddylation.** (A) Cul4A neddylation partly depends on DCNL1 expression. Western blot analysis of lysates from U2OS cell lines stably expressing siRNA-resistant transgenes for FLAG–empty, FLAG–DCNL1 (WT), or FLAG–DCNL1 (DAD mutant). Cells were treated with control or DCNL1 siRNA for 72 h. Transgene expression was induced by doxycycline (0.5 ng/ml) for 24 h. Where indicated, MLN4924 was added for 3 h at 3 μM. Cell lysates were resolved by SDS-PAGE and immunoblotted with anti-Cul4A antibody to assess the fraction of neddylated cullin. siRNA depletion of DCNL1 reduces Cul4A neddylation, which can be rescued by re-expression of DCNL1 (WT) but not cullin-binding-deficient DAD mutant. (B) Cul4A neddylation partly depends on DCNL5. Whole cell lysates were prepared as in A but cell lines stably expressed FLAG–empty, FLAG–DCNL5 (WT) or FLAG–DCNL5 (DAD mutant) and siRNA was targeted against DCNL5 or control. siRNA-mediated depletion of DCNL5 reduces the fraction of Cul4A that is neddylated. Upon re-expression of WT DCNL5, Cul4A neddylation is rescued to wild-type levels. The cullin-binding-deficient DAD patch mutant of DCNL5 fails to rescue Cul4A neddylation, suggesting DCNL5 directly interacts with Cul4A to promote neddylation. (C) Cul4A neddylation in DCNL1-KO cells directly depends on DCNL1 expression. Cul4A neddylation was assessed by western blot analysis of lysates from U2OS DCNL1 KO cell lines that stably express Flp-In-generated GFP, GFP–DCNL1 (WT) or GFP–DCNL1 (DAD mutant). Similar to the Cul4A neddylation reduction by siRNA depletion of DCNL1, knockout of DCNL1 can only be rescued by re-expression of WT DCNL1, but not DAD patch mutant DCNL1. (D) Cul4A neddylation in DCNL5-KO cells directly depends on DCNL5 expression. Same as in C except the U2OS knockout cell line was DCNL5 and rescue lines were Flp-In for GFP, GFP–DCNL5 (WT) or GFP–DCNL5 (DAD mutant). Cul4A neddylation is rescued by re-expression of WT but not DAD patch mutant DCNL5. (E) DCNL5 depletion has an additive effect on Cul4A neddylation in DCNL1 KO-cells. The fraction of neddylated Cul4A was determined by western blot analysis of cell lysates from WT or DCNL1 KO treated with control siRNA or siRNA against DCNL5. Additional depletion of DCNL5 in DCNL1-KO cells further reduces the amount of neddylated Cul4A. This additive effect of DCNL5 depletion suggests there are separate pools of Cul4A that are independently neddylated by DCNL1 or DCNL5. (F) DCNL1 depletion has an additive effect on Cul4A neddylation in DCNL5-KO cells. Same as in E, but the cell lines were WT or DCNL5 KO and treatment was with control siRNA or siRNA against DCNL1. The additional depletion of DCNL1 in DCNL5-KO cells mirrors the result in E and further supports that model that separate pools of Cul4A are independently regulated by DCNL1 or DCNL5. All graphs plot the means±s.e.m. of quantified percentage of neddylated Cul4A. Statistical significance was determined by one-way ANOVA with Newman–Keuls multiple comparison test. **P≤0.01, ***P≤0.001; *n*≥3. Asterisks denote the slower migrating neddylated form of the two cullin-reactive bands.

To test whether this was the case, we more closely examined the effect of DCNL1 and DCNL5 knockout on Cul4A, as both knockout cell lines showed a reduction of Cul4A neddylation (Fig. 4C). This reduction, albeit mild, could be rescued by re-expressing wild-type DCNL1 or DCNL5 but not DAD patch mutant forms (Fig. 5A–D). Additional siRNA-mediated knockdown of DCNL5 in the DCNL1 knockout cell line, or DCNL1 in the DCNL5 knockout cell line, further reduced Cul4A neddylation levels, which suggested that DCNL5 and DCNL1 act independently from each other to neddylate distinct Cul4A pools (Fig. 5E,F). However, it is unclear which Cul4A pools are affected by DCNL1 or DCNL5 and whether they have distinct functions. Cul4A has been implicated in processes both in the cytoplasm (Kuang et al., 2013) and in the nucleus (Chen et al., 2001; Nag et al., 2001; Shiyanov et al., 1999), where Cul4A is involved in DNA replication and DNA repair (Zhong et al., 2003; Higa et al., 2003; Hu et al., 2004). CRL4 complexes (comprising Cul4A, RBX1 and DDB1) are particularly important for the management of UV-induced DNA lesions and cells that lack the CRL4 substrate receptor DDB2 are very sensitive to UV light (Chu and Chang, 1988; Scrima et al., 2008; van Cuijk et al., 2015).

**Fig. 6.**
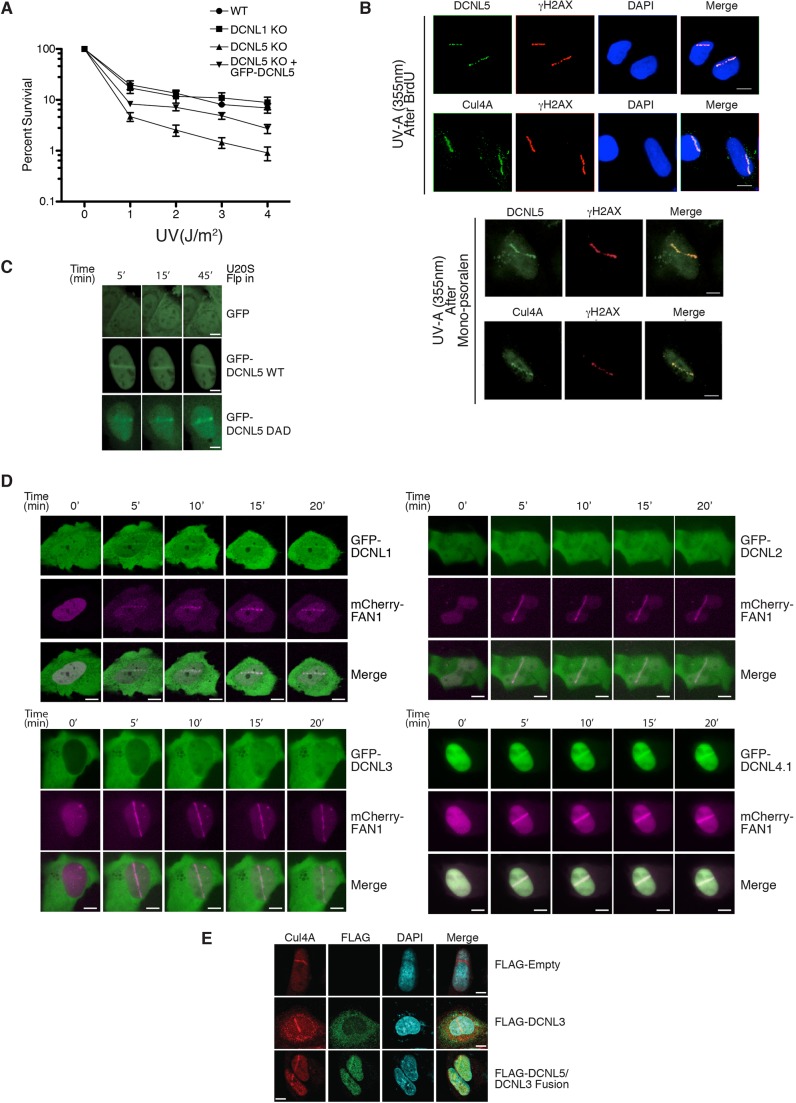
**DCNL5 but not DCNL1 is involved in the DNA damage response.** (A) Clonogenic survival analysis of U2OS cells (WT, DCNL1 KO, DCNL5 KO and DCNL5 KO re-expressing WT DCNL5) carried out after UV exposure. 500 cells/well were plated in six-well plates and 24 h later exposed to indicated amounts of UV radiation. Cells were allowed to grow for 14 days and colony formation was assessed by Crystal Violet staining. Cells able to form colonies are interpreted as having repaired the UV-induced damage. DCNL5-KO cells have an impaired ability to form colonies after UV exposure, suggesting defects in DNA repair mechanisms. DCNL1-KO cells are not sensitive to DNA damage. Each datapoint is the mean±s.e.m. of three experiments undertaken in at least three replicates. (B) DCNL5 is recruited to sites of DNA damage. Immunofluorescence of U2OS cells treated with BrdU (10 µM, 24 h; upper panels) or mono-psoralen (25 µM, 3 h; lower panels). Cells were fixed 5–10 min after laser micro-irradiation and stained for endogenous DCNL5 or Cul4A, phosphorylated γ-H2AX and DAPI. The histone variant γ-H_2_AX, a marker of DNA damage, serves as a positive control. DCNL5 and Cul4A are also recruited to the site of DNA damage. (C) DCNL5 is recruited to DNA damage sites independently of cullin binding. Live-cell analysis of U2OS cells stably expressing GFP, GFP–DCNL5 (WT) or GFP–DCNL5 (DAD mutant). Cells were BrdU-treated (10 µM, 24 h) and images captured at indicated times after micro-irradiation with a 355 nm UV laser. Both WT DCNL5 and mutant DCNL5 are recruited to DNA damage sites. (D) DCNL recruitment to sites of DNA damage. U2OS cells were transfected with N-terminally GFP-tagged DCNLs. 24 h after transfection, cells were treated as in C. All slides were treated identically and images were taken with the same microscope with identical settings. All DCNLs are readily recruited to sites of DNA damage with the exception of DCNL3, which displays only very weak recruitment. (E) U2OS cells transiently transfected with FLAG–DCNL3 or chimeric FLAG–DCNL3 where the N-terminal domain (aa 1–85) was replaced with the N-terminus domain of DCNL5 (aa 1–46). Cells were treated as in C and D. Replacement of the N-terminus readily targets DCNL3 to sites of DNA damage. Scale bars: 10 µm.

Given that DCNL1 and DCNL5 affect Cul4A neddylation, we asked if they are essential for CRL4-dependent DNA damage repair. To test this, we determined sensitivity of the knockout cell lines in clonogenic survival assays following UV exposure. We found that only DCNL5 knockout cells showed sensitivity to UV light (Fig. 6A) that could be rescued by re-expressing GFP-tagged DCNL5 (Fig. 6A). Thus, DCNL5 knockout sensitizes cells to UV damage, which represents a function that is not shared with DCNL1.

To determine if DCNL5 is involved directly in the DNA damage response, we asked whether DCNL5, like Cul4A, localizes to sites of DNA damage. We sensitized cells with 5-bromo-2′-deoxyuridine (BrdU) or mono-psoralen and subsequently micro-irradiated the nucleus with a 355-nm UV-A laser to induce DNA lesions along a defined track (Lachaud et al., 2014; Perez-Oliva et al., 2015). As previously reported, Cul4A localizes to this type of DNA damage (Fig. 6B) (Meir et al., 2015) and, importantly, endogenous DCNL5 does as well (Fig. 6B). An exogenously expressed GFP–DCNL5 construct was also recruited to the track of DNA damage independently of the interaction with cullins, as the GFP–DCNL5-DAD patch mutant was similarly recruited (Fig. 6C). In DCNL5-DAD patch mutant cells, Cul4A was also still recruited to sites of damage, demonstrating that the ability of DCNL5 to bind to cullins is not required for the localization of Cul4A (Fig. S4A). Thus, we concluded that the sensitivity of DCNL5 knockout cells to UV induced damage is most likely a direct consequence of its role in neddylating Cul4A at sites of damage.

Because of the lack of UV sensitivity of DCNL1 knockout cells, we expected that DCNL5 would be the only DCNL recruited to damage sites. To confirm this assumption, we also tested whether any of the other DCNLs localized to sites of damage. Contrary to our expectations, we found that all DCNLs were strongly recruited (Fig. 6D), with the exception of DCNL3 that was barely detectable at damage sites. It thus appears that most DCNLs might have a role at sites of DNA damage. The significance of this finding, however, is unclear at the moment. There might be redundancy in the system, but given that the DCNL5-knockout cells show sensitivity to UV-induced DNA damage, whereas the DCNL1-knockouts do not, it is likely that at least the role of DCNL5 at damage sites is to some degree non-redundant.

DCNL3 is the only DCNL that does not seem to readily go to sites of damage. However, even DCNL3 might regulate the DNA damage response, as siRNA to DCNL3 leads to increased Cul4A neddylation. Whether this effect is indirect or direct, by for example DCNL3-dependent sequestration of Cul4A in the cytoplasm, is unclear. However, as overexpression of DCNL3 does not lead to a defect in the recruitment of Cul4A to sites of DNA damage or to decreased neddylation of Cul4A (Fig. 6E; Fig. S4B), it is unlikely that the effect is simply mediated by means of sequestration. Furthermore, when we replaced the N-terminus of DCNL3 with that of DCNL5, DCNL3 was equally well recruited to sites of damage as other DCNLs; thus, any unique function of DCNL3 is mediated by its N-terminus (Fig. 6E), which based on our results, is probably true for the other DCNLs as well.

## DISCUSSION

Cullin RING ligases are the largest class of ubiquitin E3s and because of their modularity are able to form complexes that target hundreds of substrates for ubiquitylation. This class of E3 ligases share a common regulatory mechanism involving neddylation of the central cullin subunit, which is mediated in part by the DCNLs. It has long been puzzling why higher organisms contain multiple DCNLs that differ in their N-terminal domains. The most obvious explanation would be that they target different cullin subtypes, allowing the cell to regulate cullins independently. However, this is not the case, as DCNLs show no obvious cullin preference in cells or *in vitro*, even though small differences in affinities exist. The effects we see on the neddylation of different cullins after DCNL downregulation does, however, not directly correlate with the reported differences in affinities (Monda et al., 2013) or our observed slight differences in interaction between cullins and DCNLs as analysed by mass spectrometry. Other factors thus probably govern which cullin is affected by a specific DCNL. For instance, given that we found that most DCNLs are in complex with inactive cullin–RING cores and CAND1, it is hard to predict which DCNL interacts with the more ‘active’ subpopulation of any given cullin. For example, although DCNL1 might immunoprecipitate overall more Cul5 than DCNL5, it is possible that most DCNL1-bound Cul5 is inactive and also bound to CAND1, whereas the DCNL5-bound Cul5 is more readily engaged with active complexes. If so, then inactivation of DCNL5 would affect neddylation of Cul5 more than inactivation of DCNL1, even though more DCNL1 co-precipitates with Cul5.

So what are the functions of the different DCNLs and how are they regulated? Importantly, we now show that in cultured cells the DCNL isoforms display different subcellular localization patterns. It is thus feasible that through regulation of different DCNL subtypes, the cell specifically regulates cullin–RING ligase activity in different places in the cell. Our data further suggests that compartmentalization is mediated by the N-termini, which probably have regulatory functions independent of the actual NEDD8 E3 activity of DCNLs. Furthermore, studies on DCNL3 have shown that its N-terminus is important for localization to the plasma membrane, where it neddylates Cul3 (Meyer-Schaller et al., 2009). We also find DCNL3 at the plasma membrane, but curiously, downregulation of DCNL3 by siRNA increases the neddylation of many cullins. Thus, at least in some instances, DCNL3 appears to inhibit cullin neddylation (Huang et al., 2014). The mechanism by which this occurs, however, remains elusive, but we can speculate that DCNL3 sequesters cullins away from the neddylation machinery.

One surprising result was that all DCNLs strongly interact with CAND1, which was counterintuitive, as CAND1 only binds non-neddylated cullins (Liu et al., 2002; Zheng et al., 2002). However, our data unequivocally demonstrate that DCNLs and cullin–RBX1 form heterotrimeric complexes with CAND1. CAND1 binding prevents cullin neddylation by DCNLs, which is counteracted by substrate adaptors. The presence of a NEDD8 E3 in a non-neddylatable CRL might be important to allow a rapid activation when the need arises. These complexes thus probably represent inactive cullin reservoirs primed for activation by neddylation. Given that we find that DCNLs only interact with non-neddylated cullins, it is likely that DCNLs dissociate upon neddylation and that in the cell most DCNLs are in fact present in inactive CRL complexes.

Our data also strongly suggest that all DCNLs contribute to the neddylation of most, if not all, cullins and it is thus likely that any cullin can be targeted by any DCNL as long as it is present in the same compartment. The system is further complicated by the fact that the DCNLs show some overlap in localization. For example, DCNL1 and DCNL2 are both present in the nucleus and cytoplasm, DCNL3 localizes to the plasma membrane, whereas DCNL4 and DCNL5 are exclusively nuclear. So theoretically, all DCNLs should be able to neddylate nuclear cullins. However, there are still functional differences, as our data suggest that DCNL5 serves a unique role in the DNA damage response even though other DCNLs also localize to sites of DNA damage. Future experiments will be required to determine their role, but given the lack of sensitivity of DCNL1 knockout cells to UV light, it is possible that DCNL1 acts redundantly with one or all of the other DCNLs at damage sites, whereas DCNL5 retained a unique function. Similar to our findings, it was recently shown that the NEDD8 E2s UBE2M and UBE2F both localize to laser stripe, but only UBE2M depletion made cells sensitive to DNA damage (Brown et al., 2015).

In general, all our data is consistent with a model whereby, depending on its localization, a cullin becomes activated by a different DCNL. As a consequence, the overall neddylation of the cellular pool of a cullin would not be strongly affected by depletion of single DCNLs, which is what we observed. Furthermore, depletion of two DCNLs has additive effects on cullin neddylation, which further supports the idea that different DCNLs are neddylating different subpopulations of the same cullin. This ‘specialization’ might allow the cell to regulate the activity of distinct cullin pools without affecting all CRLs built around one cullin subtype.

Furthermore, all DCNLs are widely expressed in tissue culture cells, which is very different from the organismal level where tissue-specific expression is apparent. This ectopic expression of most DCNLs might thus contribute to some degree to the redundancy of the system in tissue culture. Moreover, given that at least DCNL1 and DCNL5 are oncogenic, it is crucially important for the cell to regulate the activity of DCNL E3s. Although speculative, it is possible that strong DCNL1 and/or DCNL5 expression interferes with regulatory mechanisms on other DCNLs, overriding their regulation and facilitating the development of cancer.

Given these results, it will become important in the future to study these enzymes in defined primary cells and relevant physiological contexts to understand their specific functions. This will especially be important for the DCNLs that show tissue-specific expression. These NEDD8 E3s will probably have unique functions in these tissues that are impossible to study in cancer cell lines. To fully understand the specificity, regulation and function of all DCNLs will thus require a closer look at tissue-specific roles and the generation of animal models that carry deletions or mutations of single DCNLs.

## MATERIALS AND METHODS

### Antibodies

Immunoblotting: anti-FLAG M2 (F3165, Sigma, 1/2000); anti-DCNL1 (Clone 3D7, Sigma-Aldrich, 1/1000); anti-GAPDH (Cell Signalling Clone 14c10 cat. no. 2118, 1/2000); anti-Actin (MAB1501, Millipore, 1/1000); anti-cullin1 (718700, Life Technologies, 1/1000); anti-cullin2 (700179, Life Technologies, 1/5000); anti-ACADVL (PA5-29959, Thermo Scientific, 1/1000); mouse anti-GFP (ab184519, Abcam, 1/2000). Sheep polyclonals were raised against full-length KLHL3, the N-terminus of DCNL3, 4 and 5, Cul4A and 4B, and against the C-terminus of Cul3 and Cul5, and used at 1 µg/ml.

Immunofluorescence: mouse anti-FLAG M2 (F3165, Sigma, 1/1000); chicken anti-GFP (ab13970, Abcam, 1/1000); sheep anti-DCNL5 (our own 1/200); sheep anti-cullin4A (our own, 1/100); anti-γ-H2Ax (05-636, Millipore, 1/1000), and secondary antibodies conjugated to Alexa-Fluor-488 or -594 from Life Technologies (1/1000).

### Cell culture

U2OS and HEK-293 were grown in GIBCO DMEM with 10% GIBCO FBS, L-glutamine, 100 units/ml penicillin and 100 µg/ml streptomycin (Life Technologies).

HEK-293 and U2OS cells stably expressing tagged DCNLs were generated using the Flp-In T-Rex system (Life Technologies). Expression was induced with 1 µg/ml tetracycline (Sigma-Aldrich) overnight. All cell lines used were originally obtained from ATCC and regularly tested for contamination.

### Cell extracts, immunoprecipitation and immunoblot analyses

Whole-cell extracts were prepared by lysis in 50 mM Tris-HCl pH 7.4, 150 mM NaCl, 1 mM EDTA, 1 mM EGTA, 50 mM sodium fluoride, 5 mM sodium pyrophosphate, 10 mM sodium β-glycerol-1-phosphate, 1 mM sodium orthovanadate, 0.27 M sucrose, 1% Triton X-100, 15 mM iodoacetamide, 3 mM 1,10 phenanthroline (Sigma-Aldrich) and complete phosphatase inhibitor PhosSTOP (Roche). FLAG-tagged proteins were isolated by immunoprecipitation from 2–3 mg of lysate using 10 µl FLAG(M2)-magnetic beads (Sigma) for 2 h at 4°C.

Immunoprecipitations were washed in lysis buffer before elution by LDS sample buffer. To detect protein in cell lysates, samples were separated by SDS-PAGE, transferred onto nitrocellulose or PVDF and visualized by immunoblotting or ECL (Millipore).

### RNA interference

Transfection was carried out using Lipofectamine as described previously (Hjerpe et al., 2012a,b). SMARTpool siRNA oligos from GE Dharmacon (Little Chalfont, UK) were used, siRNA against the DCNL1 3′UTR (5′-UACAUAGUCUGUACAAUAA-3′) was synthesized by Eurofins (Ebersberg, Germany).

### Cas9/CRISPR knock out cell lines

The guideRNA vectors for exon 1 in DCNL1 and DCNL5 were generated by mutagenesis PCR of pEsgRNA (Munoz et al., 2014). The target sequences for DCNL1 and DCNL5 were respectively CCAACATGGTGAGGCACTGCGGC and GCAGCAGTAGCGGAAGACGGAGG (+ strand 5′–3′). The constructs were transfected using GeneJuice (Millipore) into U2OS stably expressing FLAG–Cas9 under tetracycline-inducible promoter and single-cell cloned.

### Immunofluorescence

Cells were fixed (10 min, 4% paraformaldehyde), permeabilized (5 min; 0.5% NP40 in PBS; room temperature); blocked overnight [4°C; 3% IgG-free BSA (Jackson ImmunoResearch) and 0.02% Tween in PBS]. Cells were stained with anti-FLAG (1:2000) followed by anti-mouse Alexa Fluor 488 (1:1000), Rhodamine-conjugated phalloidin (Life Technologies, 1/4000) and DAPI (Sigma-Aldrich; 1 µg/ml), and mounted using Mowiol 4-88 (Polysciences Inc., Warrington, PA, USA).

### Protein expression and purification

The following purified proteins were previously described in Kelsall et al., (2013): Nedd8 and the APPBP1–UBA3 heterodimer (Ohta et al., 2013); DAC–TEV–Cul3–RBX1 and KLHL3 (Schumacher et al., 2015); GST–CAND1. DCNL1, 2, 3, 4 and 5 were expressed with His_6_ tags in BL21 and purified by Ni^2+^–Sepharose (GE Healthcare). GST–DCNL1 and GST–DCNL2 were purified by GSH–Sepharose (GE Healthcare).

### *In vitro* binding assays

3.6 µg His_6_–DCNL was bound to magnetic Ni-NTA beads (Sigma) at 4°C in 50 mM Tris-HCl pH 8.0 and 20% glycerol. Cul3–Rbx1–CAND1 complexes were formed by incubating 9.2 µg Cul3–Rbx1 with 12.4 µg CAND1 at 4°C. His_6_–DCNL beads were incubated with Cul3–Rbx1–CAND1 for 20 min at 4°C, then washed three times with binding buffer containing 10 mM imidazole (Sigma). Complexes were eluted with 300 mM imidazole and analysed by SDS-PAGE and Coomassie staining.

### Neddylation assays

Cul3–Rbx1 (1 µM) and 1 µM DCNL were incubated with 34 µM NEDD8, 4 µM UBE2M and 0.2 µM NAE in reaction buffer (50 mM Tris-HCl pH 8.0, 0.15 mM ATP, 1.5 mM MgCl_2_, 20% Glycerol) for 2 min at 30°C, then quenched with LDS buffer. In some reactions 1 µM CAND1 or 1 µM CAND1 plus 10 µM KLHL3 were included.

### Laser irradiation and confocal microscopy

U2OS cells were seeded in 35-mm glass-bottom dishes at 1×10^6^ cells per dish and incubated with mono-psoralen at 25 µM for 2 h or with BrdU at 10 µM for 24 h. A PALM MicroBeam system (Carl Zeiss AG, Oberkochen, Germany) was used to irradiate with the 355 nm UV laser at 20–25% for cells treated with mono-psoralen and BrdU. Indirect immunofluorescence was performed as described previously (Lachaud et al., 2014). A minimum of 100 cells were irradiated per replicate.

### Induction of DNA damage and colonogenic cell survival assays

500 cells/well were plated in six replicates onto 6-well plates. Media from cells was aspirated 24 h after plating and cells were exposed to UV using a Spectrolinker XL-1500 UV cross-linker (Spectroline, Westbury, NY). Fresh media was added and colonies were grown for 14 days. Media was aspirated and plates stained with Crystal Violet (Sigma). Colonies with >100 cells were counted. For each condition, cell viability of untreated cells was defined at 100%.

### Mass spectrometry analysis

After immunoprecipitation and washings with lysis buffer and detergent-free buffer (100 mM Tris HCl pH 8.5), FLAG-tagged proteins were eluted with acetonitrile/formic acid 1% (ratio 1:1). The eluate was dried (Speedvac, Thermo Scientific) and resupended in 300 µl buffer containing 1 M urea, 100 mM ammonium bicarbonate pH 8.0, 5 mM DTT (Sigma-Aldrich), and 0.01% RapiGest (Waters, Milford, MA). 5% of eluate was processed by SDS-PAGE on 4–12% gradient gels and silver stained. The remainder was alkylated in the dark for 60 min at 30°C in 15 mM chloroacetamide (Sigma-Aldrich). Samples were digested overnight at 37°C with 3.75 µg/ml of mass-spectrometry-grade Trypsin (Promega; Madison, WI). Samples were acidified to pH 3 (trifluoroacetic acid; Sigma-Aldrich) and centrifuged at 17,000 ***g*** for 5 min to remove RapiGest. Supernatant was bound to c18 columns (Harvard Apparatus; Holliston, MA) washed with 0.1% TFA and eluted with 60% acetonitrile and 0.1% TFA. Samples were run on a Thermo Scientific Orbitrap Classic. RAW files were analysed with MaxQuant and interaction heatmaps were generated in R (R-project).
